# Identification of Novel Transcriptional Alleles in Primary Prostate Cancer Cells and Cancer Stem Cells by Bulk RNA-Seq and Single-Cell RNA-Seq Analyses

**DOI:** 10.3390/biom16091341

**Published:** 2026-09-15

**Authors:** Wen-Yang Hu, Ranli Lu, Mark Maienschein-Cline, Duoling Xu, Parivash Afradiasbagharani, Lynn A. Birch, Larisa Nonn, Andre Kajdacsy-Balla, Toshi Shioda, Gail S. Prins

**Affiliations:** 1 Department of Urology, College of Medicine, University of Illinois Chicago, Chicago, IL 60612, USA; ranlilu0424@gmail.com (R.L.); duoling@uic.edu (D.X.); pafrad2@uic.edu (P.A.); lbirch@uic.edu (L.A.B.); 2 Research Resources Center, Office of the Vice Chancellor for Research, University of Illinois Chicago, Chicago, IL 60612, USA; mmaiensc@uic.edu; 3 Department of Pathology, College of Medicine, University of Illinois Chicago, Chicago, IL 60612, USA; lnonn@uic.edu (L.N.);; 4 Center for Cancer Research, Massachusetts General Hospital, Harvard Medical School, Charlestown, Boston, MA 02129, USA; shioda@helix.mgh.harvard.edu

**Keywords:** prostate cancer, somatic mutation, RNA-seq, cancer stem cells, single-cell analysis

## Abstract

Genetic alterations are closely associated with prostate cancer development and progression, but the RNA-derived transcriptional allele landscape of prostate cancer and cancer stem cells (CSCs) remains poorly understood. To characterize cancer-associated transcriptional alleles, we analyzed bulk and single-cell RNA sequencing (RNA-seq) data from primary human prostate cancer cells and their matched benign epithelial cells from non-cancerous regions of the same patients. Sequencing reads were aligned to the human reference genome (hg38) using STAR, and sequence variants were annotated with ANNOVAR. Most detected transcriptional alleles were located in noncoding regions, particularly within 3′ and 5′ untranslated regions, with single-nucleotide variants predominating and C>T substitutions occurring most frequently. Comparison of cancer and matched benign samples identified 223 genes carrying cancer-associated transcriptional alleles consistently detected across three independent patients. Seven of these genes showed differential expression between CSC and non-CSC populations, while 19 contained non-synonymous alleles, including 11 that are predicted to have potentially damaging effects. We identified ATF6 and KDM3A as candidate genes of potential functional interest. Single-cell RNA-seq analyses revealed differences in the number and distribution of detected transcriptional alleles between culture conditions, with 2D cancer cultures detecting more total, coding, and non-synonymous alleles than CSC-enriched 3D spheroids. CSC populations also exhibited fewer detected transcriptional alleles than non-CSC populations. Our findings provide a framework for characterizing transcriptional allele heterogeneity within the prostate cancer cellular hierarchy and identify candidate CSC-associated alterations for further investigation and functional validation. Finally, the cancer–benign matched strategy used in this study provides additional molecular evidence supporting the malignant origin of the tumor-derived cancer cells.

## 1. Introduction

Genetic testing is recommended for individuals with a personal or family history of certain cancers to assess inherited cancer risk. Identification of pathogenic mutations can guide therapeutic decision-making and inform the need for earlier and more intensive screening, particularly among individuals with strong familial predisposition. Although most prostate cancers arise sporadically, specific inherited genetic alterations substantially increase lifetime risk and are often associated with more aggressive disease phenotypes [1,2,3]. Germline alterations in DNA-repair genes are estimated to contribute to approximately 10% of prostate cancer cases, with an even higher prevalence observed in early-onset and metastatic disease [4,5]. Large-scale genomic studies have further demonstrated that alterations affecting DNA-repair pathways, androgen receptor signaling, and chromatin regulation are important features of prostate tumorigenesis and progression. Comprehensive genomic profiling studies, including those by The Cancer Genome Atlas Research Network and the metastatic prostate cancer analysis led by Robinson and colleagues, have demonstrated extensive genomic heterogeneity and recurrent alterations in genes such as *BRCA2*, *ATM*, *SPOP*, and *FOXA1* [5,6,7,8]. These findings underscore the importance of defining the molecular landscape of prostate cancer at high resolution and identifying additional cancer-associated sequence alterations.

While most large-scale studies have relied primarily on DNA-based approaches, transcriptomic RNA sequencing (RNA-seq) provides complementary information by simultaneously capturing gene expression and sequence variation within actively transcribed regions. RNA-seq can identify expressed coding and noncoding sequence alterations, splice-associated changes, allele-specific expression, and other transcriptional events that may contribute to cancer phenotypes. RNA-derived sequence variants may also provide information complementary to DNA-based datasets, as they reflect alterations that are actively transcribed in tumor cells. Recent pan-cancer studies have demonstrated the utility of RNA-seq for identifying expressed sequence variants and expanding the characterization of cancer-associated molecular alterations [9,10]. However, RNA-seq–derived variants cannot necessarily be interpreted as genomic somatic mutations. RNA editing, allele-specific expression, alternative splicing, rare germline variation, transcript abundance, sequencing coverage, and mapping artifacts can all influence the detection of RNA-derived sequence variants. Therefore, sequence alterations identified exclusively from RNA-seq are more appropriately considered transcriptional alleles unless their genomic origin is independently confirmed by DNA sequencing. In the current study, we analyzed both bulk RNA-seq and single-cell RNA-seq (scRNA-seq) datasets derived from human prostate cancer cells and their matched benign epithelial cells to characterize cancer-associated transcriptional alleles. Bulk RNA-seq analysis was performed on matched samples from three independent patients, and single-cell RNA-seq was used to examine the transcriptional allele patterns across tumor cell populations, including CSC-enriched 3D spheroids and non-CSC populations. We systematically identified and annotated RNA-derived sequence variants and examined their distribution across coding and untranslated regions, variant classes, and nucleotide substitution patterns. We further focused on genes carrying cancer-associated transcriptional alleles that were reproducibly detected across the three independent patient samples and integrated these findings with single-cell gene-expression profiles to explore their associations with CSC populations. Another advantage of the cancer–benign matched strategy is that, in addition to identifying candidate cancer-associated RNA-derived transcriptional alleles, it provides independent molecular evidence that distinguishes tumor-derived epithelial cells from matched benign epithelial cells, addressing a longstanding challenge in verifying the malignant identity of cells isolated from patient specimens.

Using this approach, we identified 223 genes harboring cancer-associated transcriptional alleles consistently detected across three independent patients. Among these genes, 19 contained non-synonymous transcriptional alleles, including 11 genes predicted to be functionally damaging. Single-cell RNA-seq analysis also revealed differences in the number and distribution of detected transcriptional alleles across culture conditions, with fewer alleles detected in CSC-enriched 3D spheroids than in their corresponding non-CSC populations. Because the identified alleles were derived from RNA-seq without orthogonal DNA-based validation, they may reflect genomic mutations, RNA editing, allele-specific expression, or other transcriptional and technical effects. Therefore, they are interpreted as differences in RNA-derived transcriptional allele detection rather than direct evidence of differences in genomic mutation burden or genomic stability. Overall, this study provides a proof-of-concept transcriptomic approach to identify cancer-associated transcriptional alleles in prostate cancer and generate candidate alterations and genes for subsequent validation using DNA-based sequencing and functional studies.

## 2. Materials and Methods

### 2.1. Prostate Epithelial Cell Cultures

Primary human prostate epithelial cells derived from prostate cancer (PrE-Ca) and matched benign cells from the peripheral zone tissue (PrE-pz) were isolated from radical prostatectomy specimens obtained at the University of Illinois Chicago Medical Center under an Institutional Review Board–approved protocol. Fresh peripheral zone tissue was excised using a 5 mm punch by a pathologist, and adjacent sections were confirmed as either 0% or 100% cancer by hematoxylin and eosin staining [11]. PrE-Ca and PrE-pz were maintained at 37 °C, 5% CO_2_ in ProstaLife Epithelial Cell Growth Medium (PrEGM) (Lifeline Cell Technology, Frederick, MD, USA), as previously described [12,13]. PrE-Ca were derived from four patients, including African American and European American patients, with Gleason scores 3 + 3/3 + 4 and with 70–90% cancer cells.

### 2.2. Prostate Cancer Spheroid Culture

Stem cell–enriched spheroids were generated from PrE-Ca or PrE-pz as previously reported [12,13,14]. Briefly, 1 × 10^5^ cells were suspended in a 1:1 mixture of Matrigel (Corning Life Sciences, Tewksbury, MA, USA) and PrEGM and plated along the edge of 12-well plates. After gel solidification, 1 mL PrEGM was added and refreshed every 48 h. Spheroids were cultured at 37 °C in 5% CO_2_ for 7 days.

### 2.3. Bulk RNA Sequencing

Total RNA was extracted from 2D prostate epithelial cultures derived from matched prostate cancer and benign regions from three independent patients, a total of six patient specimens. Indexed libraries were prepared following cDNA synthesis and fragmentation and sequenced on the Illumina NextSeq 500 platform at the University of Illinois Biotechnology Center using paired-end 75 bp reads, yielding >50 million reads per sample. Library quality and size distribution were assessed using the (Agilent Technologies, Inc., Waldbronn, Germany). Bulk RNA-seq raw reads were aligned to the human reference genome (hg38) using STAR v2.7.11b in two-pass mode to improve splice junction detection [15]. BAM files were coordinate-sorted, and apparent PCR duplicates were marked using Picard MarkDuplicates. Only uniquely mapped reads with a mapping quality ≥30 were retained [16].

### 2.4. Single-Cell RNA Sequencing

The scRNA-seq experiment included four samples from the same prostate cancer patient: 2D cancer epithelial cells, 3D cancer spheroid cultures, 2D matched benign epithelial cells, and 3D matched benign spheroid cultures. Cancer and matched benign cells from both 2D and 3D cultures were dissociated into single-cell suspensions and processed using the Chromium Next GEM Single Cell 3′-v3 platform (10x Genomics, Pleasanton, CA, USA). Barcoded libraries were sequenced at the University of Illinois Biotechnology Center on an Illumina NovaSeq 6000 SP flow cell (paired-end 28 bp + 91 bp), generating approximately 0.1 million reads per cell. Approximately 3500 cells per sample and ~7500 genes per cell were retained for downstream analysis. Raw scRNA-seq data were processed using Cell Ranger (10x Genomics) against the GRCh38/hg38 reference genome. The filtered feature–barcode matrix was analyzed using Seurat v3. Quality control filtering removed cells with <2000 or >7500 detected genes, cells with >10% mitochondrial transcripts, putative doublets, and low-quality droplets. Data were normalized using the Log Normalize method (scale factor = 10,000). Highly variable genes (n = 2000) were identified, scaled, and subjected to principal component analysis (PCA). Significant principal components were selected using a Jackstraw-inspired resampling procedure. Cells were clustered using a graph-based approach and visualized using Uniform Manifold Approximation and Projection (UMAP v0.5.0). Cluster identities, including cancer stem cell populations, were assigned based on the expression of established prostate cancer stem cell markers, keratin profiling, and epithelial cell lineage markers, as reported previously by our laboratory and others [11,14,17]. After cluster identities were finalized, the cancer cluster numbers were added as read group (RG) tags to the BAM file generated by Cell Ranger to enable cluster-specific variant calling. A common RG tag, “benign”, was used for all cells from benign samples to allow for greater sequencing depth. The expression value of each gene was scaled so that data points were centralized around the origin and the variance across these data points was equal to 1. Significant differentially expressed transcripts across clusters of single cells were identified using Benjamini–Hochberg-corrected *p*-values (FDR) for multiple comparisons. Data were analyzed using Student’s *t*-test. Values are expressed as mean ± SEM, and *p* < 0.05 was considered significant.

### 2.5. Variant Calling

Diploid nucleotide variants were called using freeBayes with a minimum variant allele frequency of 5% (−F 0.05) and minimum coverage over all samples of 10. Variants were functionally annotated using ANNOVAR to determine genomic location, coding consequences, predicted protein impact, and population genetic annotations and frequencies from dbSNP and gnomAD [18,19]. We also obtained previously characterized RNA-editing events from REDIPortal [20,21]. Non-synonymous transcriptional alleles were further evaluated using SIFT, with scores <0.05 considered deleterious [22]. Transcriptional alleles that could be characterized as germline (based on dbSNP and gnomAD) or RNA editing (based on REDIPortal annotations or SNV substitutions) were retained in the analysis, as they may still represent novel cancer-associated allelic expression or RNA editing, respectively.

### 2.6. Cancer-Associated Variant Prioritization

We identified cancer-associated transcriptional alleles by comparing variant calls from cancer samples with those from benign samples from the same individual, selecting alleles detected only in the cancer sample. This analysis was limited to sites with an overall coverage of at least 10× across. In particular, we prioritized the discovery of novel expressed alleles in the cancer cells, based on cases where: (a) we observe expression of an allele in the cancer cells, (b) we also observe expression of the same genomic position in the benign cells, but (c) we do not see expression of the cancer-associated transcriptional allele in the benign cells. To accomplish this, we identified all alleles expressed in the benign samples using low-stringency criteria and all alleles expressed in the tumor samples using high-stringency criteria and then subtracted the lists to obtain alleles unique to the tumor. Alleles are counted as present in benign cells if they have at least 2× coverage and 3% allele frequency (low stringency). Alleles are counted as present in cancer if they have at least 5× coverage and 10% allele frequency (high stringency). Novel cancer-associated transcriptional alleles were defined as those present in cancer but not in the germline [23]. In bulk RNA-seq, novel cancer-associated transcriptional alleles were only considered if they did not match the reference genome. In single-cell RNA-seq, novel cancer-associated transcriptional alleles that matched the reference were still included in our analysis.

## 3. Results

### 3.1. Variant Identification Workflow from Bulk and Single-Cell RNA-Seq

**Figure 1:**
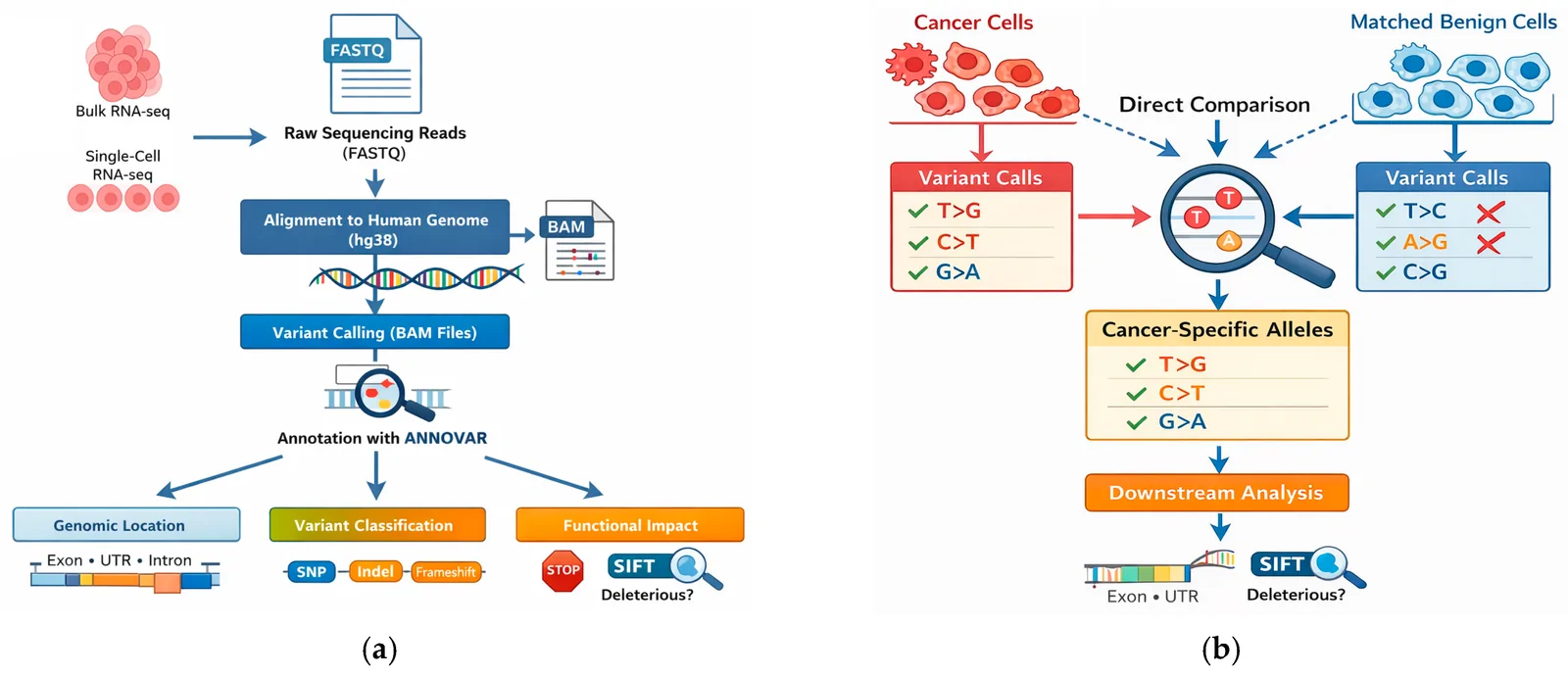
Schematic overview of the computational pipeline for identifying variant calls by RNA-seq and strategy for verification of cancer-associated alleles: (**a**) Raw sequencing reads from bulk RNA-seq and single-cell RNA-seq were aligned to the human reference genome (hg38) using STAR. Variants were called from aligned BAM files and annotated with ANNOVAR to determine genomic location, predicted functional consequences, and deleteriousness (SIFT score for non-synonymous variants). (**b**) Variant calls from prostate cancer cells were compared directly with matched benign epithelial cells derived from non-malignant regions from the same patient. Only alleles uniquely detected in cancer samples were retained for downstream analyses, as described.

To identify candidate cancer-associated transcriptional alleles in prostate cancer, we performed bulk RNA-seq and scRNA-seq on primary cultured prostate cancer cells and matched benign epithelial cells isolated from non-malignant regions of the same patients. As illustrated in Figure 1a, sequencing reads were aligned to hg38 using STAR [15], followed by variant calling and annotation with ANNOVAR [24]. Functional consequences were assigned based on gene overlap, predicted protein impact, and SIFT scores [22]. Cancer-associated transcriptional alleles were identified by directly comparing variant calls from cancer cells with those from matched benign cells from the same individual, and only alleles uniquely detected in cancer samples were retained for downstream analysis, including: (1) classification of the identified alleles according to variant type and genomic/transcriptomic location; (2) identification of genes carrying cancer-associated transcriptional alleles that were shared across the three independent patient samples; (3) analysis of the distribution of these alleles in 2D versus 3D cultures and CSC versus non-CSC populations; (4) integration of the allele-containing genes with the single-cell RNA-seq expression profiles to identify genes that were differentially expressed in the CSC population. The strategy is outlined in Figure 1b.

### 3.2. Variant Landscape Identified in Bulk RNA-Seq Data

**Figure 2:**
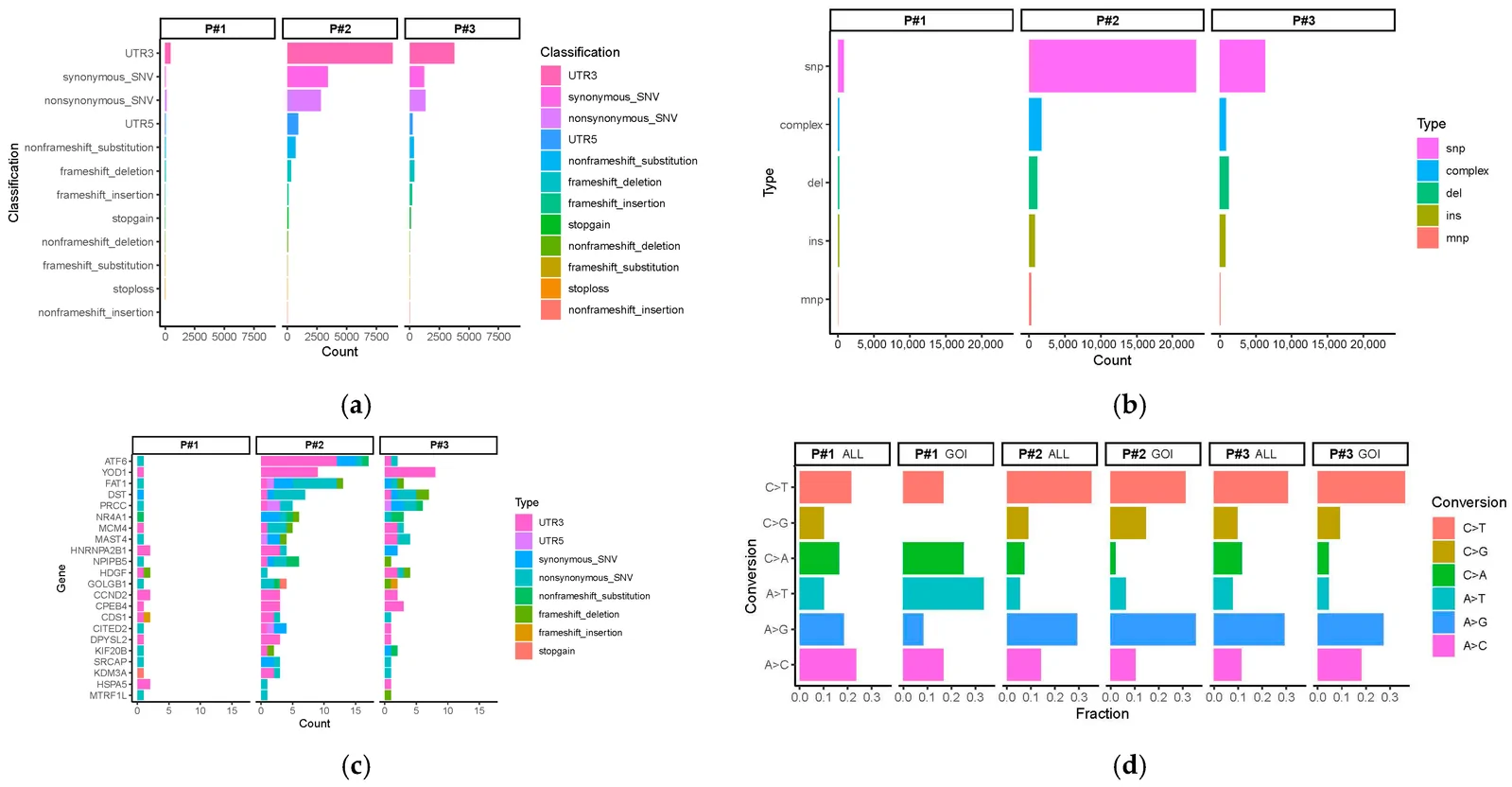
(**a**) Counts of variant types for three individual patients by bulk RNA-seq. Genomic distribution of detected variants across functional regions, showing that the majority occur in noncoding regions, particularly within the 3′ and 5′ untranslated regions (UTRs). (**b**) Classification of variant types identified in bulk RNA-seq. Predominance of synonymous and non-synonymous single-nucleotide variants (SNVs), with insertions and deletions (indels)—including frameshift-associated events—constituting a minor fraction. (**c**) Counts of variant types for the targeted genes of interest (GOI). Across all patients, the most common variant subtype is 3′ untranslated regions (UTRs), followed by synonymous/non-synonymous-SNVs and frameshift/non-frameshift variants. (**d**) SNV substitution spectra across the transcriptome and genes of interest. SNVs were classified into the six canonical base-pair substitution categories (C>T, C>G, C>A, A>T, A>G, and A>C).

To identify novel transcriptional alleles, including somatic mutations, RNA editing, and allele-specific expression, we performed bulk RNA-seq using primary cultured cells from the prostate cancer matched with benign cells from the three patients. Data analysis revealed that most variants were located and enriched in noncoding regions, particularly within the 3′ and 5′ untranslated regions (UTRs) (Figure 2a). Variant classification showed that SNVs constitute the predominant variant class, while insertions and deletions (indels)—including those predicted to cause frameshift mutations—represent a smaller fraction (Figure 2b). Counts of variant types for the relevant genes of interest (GOI) dataset showed that the majority of variant calls occur in 3′ untranslated regions (UTRs), followed by synonymous/non-synonymous single-nucleotide variants (SNVs) and frameshift/non-frameshift variants across all patients (Figure 2c). The distribution of SNV substitution types was analyzed across the entire transcriptome and within the targeted GOI for each subject. The SNV spectrum analysis encompasses the six canonical base-pair substitution classes (C>T, C>G, C>A, A>T, A>G, and A>C). Across all samples, C>T transitions were the most prevalent substitution class, consistent with spontaneous deamination of 5-methylcytosine. Due to the relatively low number of variants detected per individual GOI, SNVs were aggregated across all selected genes to enable robust characterization of substitution patterns. For GOI-level summaries, all SNVs identified in any of the selected genes were combined, as individual genes generally harbored too few variants to support meaningful per-gene comparisons (Figure 2d).

Across patients, 223 genes harboring RNA-derived unique exonic or UTR variants were detected exclusively in cancer cells (Supplementary Materials, Table S1). Among these, 19 genes contained non-synonymous variants predicted to alter protein sequence (Table 1). Of these, 11 genes were classified as functionally damaging based on variant annotation, including SIFT-predicted deleterious substitutions, frameshift mutations, stop-gain or stop-loss variants, and coding insertions/deletions (Table 2). Further, ATF6 and KDM3A were identified as candidates of potential functional interest carrying transcriptional alleles; these two genes are of particular biological relevance and have been implicated in tumor survival signaling and androgen receptor–mediated transcriptional regulation, respectively [25,26].

### 3.3. Transcriptional Allele Patterns in Single-Cell RNA-Seq

The intra-tumor heterogeneity indicates that cancers are multiclonal and derive from distinct cancer stem cells. To gain more insight into the RNA-derived transcriptional alterations in the subclusters of cancer cells, we further performed single-cell RNA-seq analysis of cancer cells derived from both primary 2D cultures and cancer stem cell–enriched 3D spheroid cultures from both the cancer and benign regions of the same patient, and our findings revealed that variant patterns shared several substitution classes despite differences in their relative frequencies with those observed in bulk RNA-seq (Table 3).

To assess the potential biological significance of these RNA-derived transcriptional alterations, we examined whether the 223 genes carrying cancer-associated transcriptional alleles were associated with stem cells and cancer progression. Among these 223 genes, seven—CITED2, YOD1, MCM4, HNRNPA2B1, KIF20B, DPYSL2, and NR4A1—were differentially expressed between the CSC and non-CSC populations by single-cell RNA-seq analysis (Table 4). One of the two CSC-enriched genes, CITED2, is often linked to cancer aggressiveness and metastasis, including in breast, colon, and prostate cancers. The statistical comparisons represent within-sample/among-cluster cell-level comparisons rather than independent patient-level biological replication. These exploratory observations require validation in additional independent patient samples.

**Figure 3:**
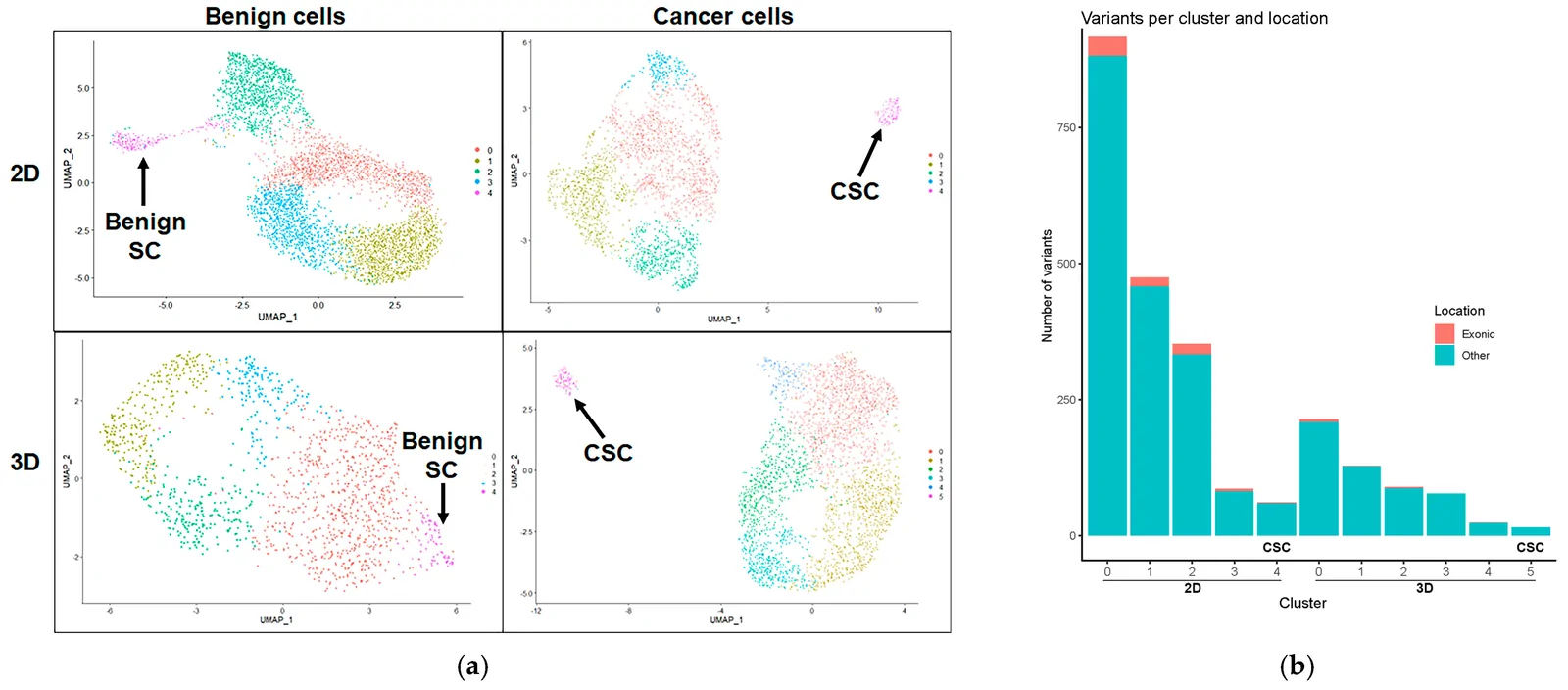

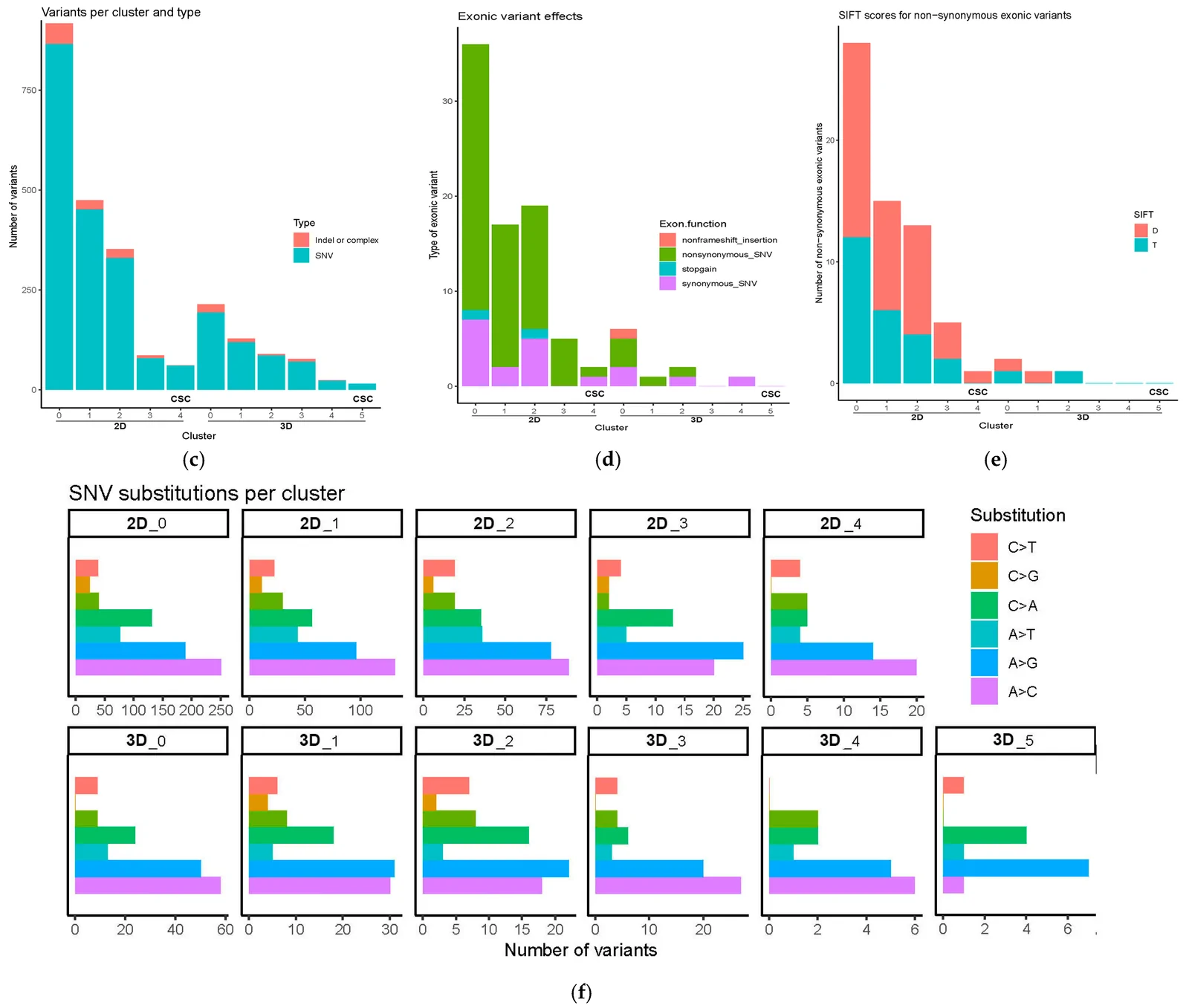
(**a**) Single-cell RNA-seq clustering analysis by UMAP. Cancer stem–like cells were identified in cluster #4 from 2D cultured prostate cancer cells and cluster #5 from 3D cultured prostate cancer organoids; cluster #4 from both 2D and 3D benign cells contains benign stem cells. (**b**) Genomic distribution of noncoding variants and exonic variants detected in prostate cancer cells from primary 2D cultures and 3D spheroid cultures. (**c**) Genomic distribution of SNVs and indels/complexes detected in prostate cancer cells from primary 2D cultures and 3D spheroid cultures. (**d**) Exonic variant subtypes detected in prostate cancer cells from primary 2D cultures and 3D spheroid cultures. (**e**) SIFT-predicted deleterious (D) and tolerated (T) non-synonymous exonic variants. (**f**) Nucleotide substitution spectrum from the entire transcriptome using single-cell RNA-seq.

Uniform Manifold Approximation and Projection (UMAP) clustering analysis identified cells in cluster #4 from both 2D and 3D prostate benign cells as benign stem cells, while cells in cluster #4 from 2D prostate cancer cells and cluster #5 from 3D prostate cancer spheroids were identified as cancer stem cells (CSCs) (Figure 3a). Prostate CSC annotation was based on the expression of established prostate cancer stem cell markers, together with our previously published single-cell analyses of CSC keratin profiling and established lineage markers, including PSCA, ALDH1A3, CEACAM6, KRT13, KRT19, SQSTM1, TACSTD2, SPRR3, S100P, and LY6D [11,17]. Across datasets, noncoding variants predominate over exonic variants (Figure 3b). Classification of variant types in cancer cells demonstrated that SNVs were the most frequent variant type, whereas insertions and deletions (indels)—including frameshift variants—were less common (Figure 3c). Clustering analysis of exonic variants in both primary 2D and 3D spheroid cultures revealed that non-synonymous SNVs are the most frequent, while synonymous SNVs, stop-gain, and non-frameshift insertions are less frequent (Figure 3d). Sorting Intolerant From Tolerant (SIFT)-predicted protein function analysis in prostate cancer cells from both primary 2D culture and 3D spheroids revealed that deleterious (D) non-synonymous SNVs are more frequent than tolerated (T) non-synonymous SNVs (Figure 3e). Further, in both the primary 2D and 3D spheroid cultures, clusters of CSCs (2D_#4 and 3D_#5) have the lowest number of exonic and non-exonic variants (Figure 3b), fewer indels/complexes and SNVs (Figure 3c), and fewer total and D/T non-synonymous SNVs (Figure 3d,e) compared to the non-CSC clusters. Finally, the spectrum of nucleotide substitutions among SNVs from single-cell RNA-seq with six canonical base-pair substitution categories (C>A, C>G, C>T, A>C, A>G, and A>T) was compared to that observed in bulk RNA-seq data, with A>C and A>G transitions being the most predominant substitution classes in non-CSC clusters in both the 2D and 3D cultures and in the 2D CSC cluster. In contrast, only the A>G transition is predominant in the 3D CSC culture (Figure 3f).

### 3.4. Reduced Number of Detected Transcriptional Alleles in Cancer Stem Cells

**Figure 4:**
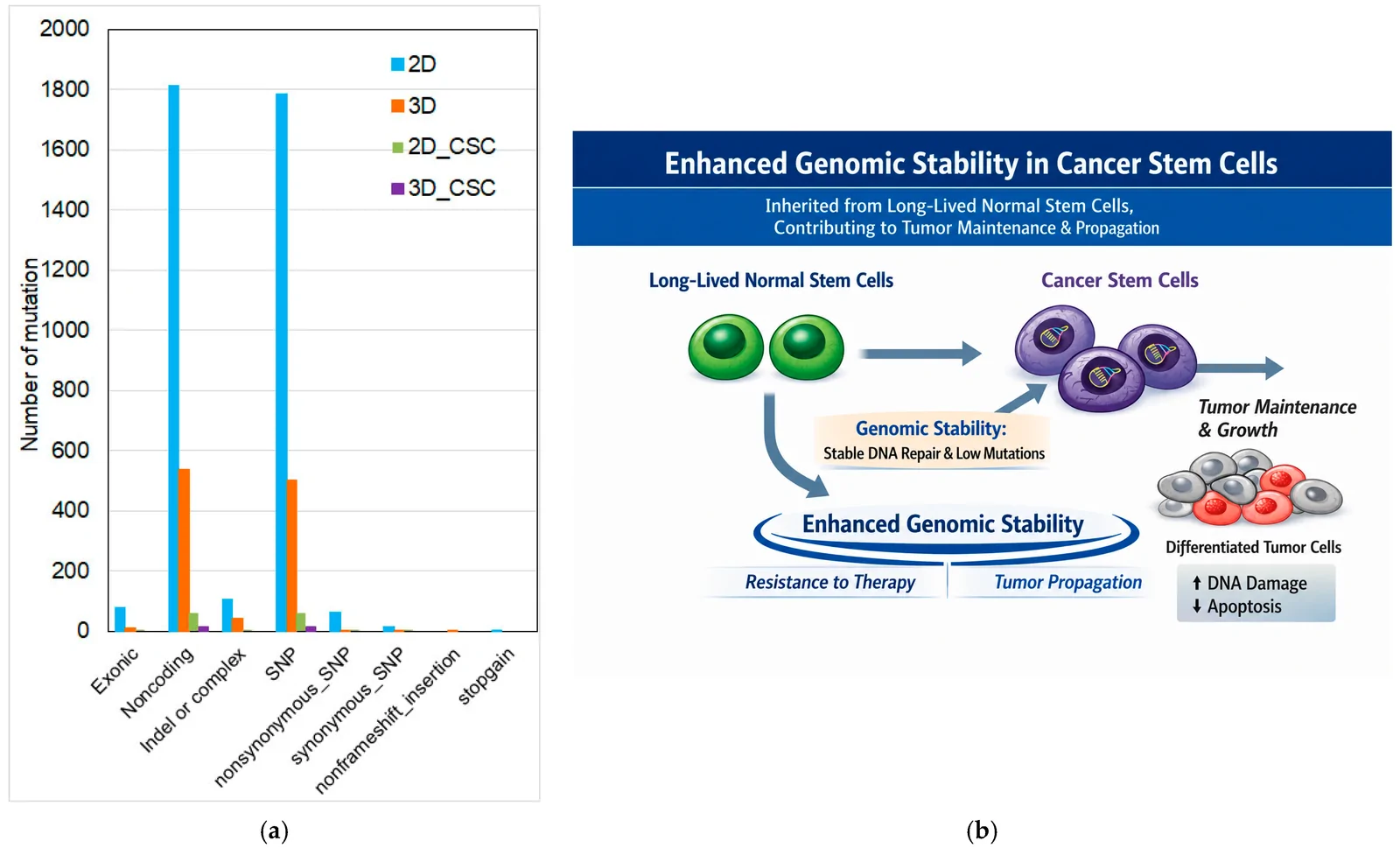
Reduced number of detected transcriptional alleles in cancer stem cells: (**a**) The numbers of variants per condition showed approximately threefold higher total detected transcriptional alleles, eightfold higher coding transcriptional alleles, and tenfold higher non-synonymous SNVs in 2D cancer cells compared with CSC-enriched 3D spheroids. Comparing the total number of variants per condition between cancer stem cells (CSCs) and non-stem cancer cells in both 2D and 3D cultures, CSCs have lower frequency of exonic transcriptional allele and the lowest number of noncoding variant events. (**b**) Proposed speculative hypothesis model illustrating one possible biological explanation that enhanced genomic stability in CSCs, potentially inherited from long-lived parental stem cells, contributes to tumor maintenance and propagation.

Cancer stem cells are frequently maintained in a relatively quiescent, slow-cycling state and possess efficient DNA damage response and repair mechanisms, which may contribute to fewer detected RNA-derived transcriptional alleles than in rapidly proliferating differentiated tumor cells. However, this interpretation remains to be validated experimentally [27,28,29,30]. In the current study, our comparative quantification analysis further demonstrated that cancer cells grown in 2D culture harbored substantially more transcriptional alleles than those in cancer stem cell–enriched 3D spheroids, including approximately threefold more total variants, eightfold more coding alleles, and tenfold more non-synonymous SNVs (Figure 4a; Table 4).

To evaluate the transcriptional allele load by cellular state, CSCs were compared with non-CSCs in both 2D and 3D cultures. Consistent with this trend, CSCs in both 2D and 3D cultures exhibited the lowest number of transcriptional alleles and fewer noncoding variant events than non-stem cancer cells (Figure 4a; Table 4).

Although the reduced number and different pattern of detected transcriptional alleles may result from differences in transcriptional activity, transcript abundance, sequencing depth, gene coverage, and technical dropout in sequencing, it also suggests a possible lower transcriptional allele burden and enhanced genomic stability in CSC populations, potentially inherited from long-lived parental stem cells and contributing to tumor propagation and persistence, as proposed in the model (Figure 4b). This model is presented as a speculative hypothesis illustrating one possible biological explanation for the observed pattern. Further investigation using matched DNA-based sequencing, quantitative mutation-burden analyses, and additional functional studies are necessary to verify the genomic stability in CSCs.

### 3.5. Evaluation of RNA-Derived Transcriptional Alleles, Germline Variants, and RNA Editing Using Public Variant Databases dbSNP, gnomAD, and REDIPortal

**Figure 5:**
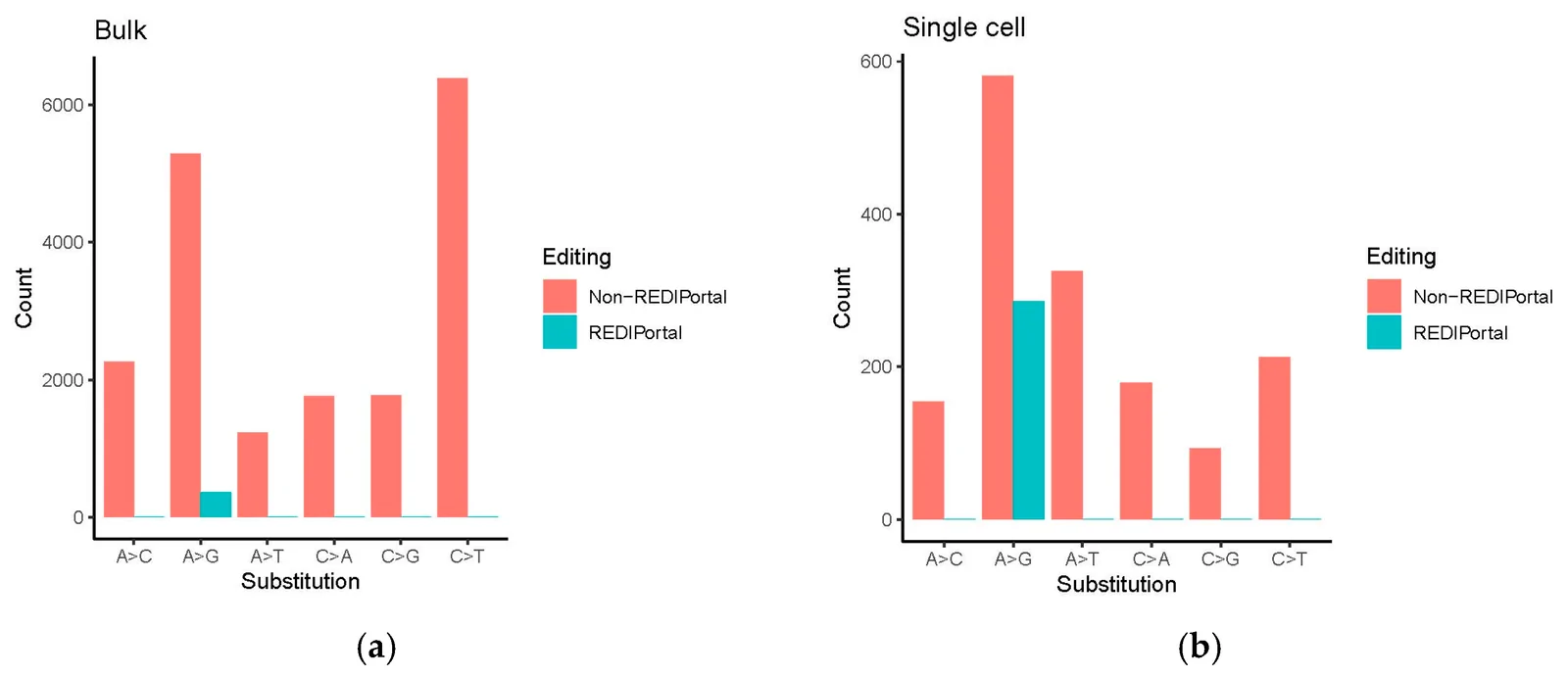
Frequency of SNV substitutions at REDIPortal sites or non-REDIPortal sites for bulk and single-cell RNA-seq: (**a**) In bulk RNA-seq analysis, there are very few annotations for RNA editing; C>T is the most common variant for non-REDIPortal sites. (**b**) In single-cell RNA-seq analysis, there is a larger percentage overlap with RNA-editing sites; note also that A>G is the most common variant for non-REDIPortal sites, so there are probably some novel RNA-editing events.

Some low-frequency variants may represent rare germline alleles rather than true somatic mutations, potentially inflating the number of putative cancer-associated variants. Without matched benign samples, distinguishing somatic mutations from rare/private germline variants is difficult [31,32]. In addition to using the cancer–benign matched strategy, we further incorporated public germline variant databases to filter common population polymorphisms. The dbSNP serves as a comprehensive global catalog for all kinds of small genetic variations with standard ID numbers [18], while gnomAD focuses specifically on high-quality population allele frequencies from large-scale sequencing projects [19]. REDIPortal is a database of characterized RNA-editing sites in human tissues [20,21]. We performed an additional analysis to identify novel alleles that may originate from germline variants or RNA editing. Based on annotations from dbSNP and gnomAD, we estimate that a minority of variants may be of germline or RNA-editing origin (Figure 5; Table 5), especially within the single-cell RNA-seq results.

For bulk RNA-seq analysis, a large number of variants are detected as unique to the tumor but overlap with common SNVs (32%). These are most likely cases of altered allelic expression in the tumor. There are very few annotations for RNA editing. It may be that editing events are more common between tumor and normal cells and are thus removed in the tumor–normal comparison step (Figure 5a; Table 5). In single-cell RNA-seq analysis, there is very little SNV overlap (3% with common SNVs, more with rare ones). We do have a larger percentage of overlap with RNA-editing sites; note also that A>G is the most common variant for non-REDIPortal sites, so there are probably some novel RNA-editing events (Figure 5b; Table 5). This suggests that individual cancer cell clusters may be differentiated partially by changes in RNA editing. We acknowledge that A>G substitutions may reflect A-to-I RNA editing and that RNA-editing events could contribute to the observed allele spectrum.

Finally, we clarified in the Methods Section that the thresholds chosen for identifying novel alleles in the tumor are weighted to require higher stringency for a positive identification in the tumor cells, and a lower stringency for a corresponding identification in the benign cells.

## 4. Discussion

Genetic alterations are central drivers of prostate tumorigenesis. Yet, the landscape of RNA transcriptional alleles within transcriptionally active regions of the genome—and its relationship to the tumor cellular hierarchy—remains incompletely defined. Distinguishing cancer-associated transcriptional alleles from inherited genetic variation represents an additional challenge. Analysis of tumor samples alone may identify both somatic and germline variants, including rare germline alleles that are not readily distinguishable from tumor-associated alterations. Patient-matched benign epithelial cells provide an important internal reference because sequence alterations detected in both tumor and matched benign cells can be excluded from the cancer-associated set. In the present study, we therefore compared prostate cancer epithelial cells with matched benign epithelial cells derived from non-malignant regions of the same patients to identify cancer-associated transcriptional alleles. This paired design provides a stringent within-patient comparison and reduces the potential contribution of patient-specific inherited variation. Nevertheless, we recognize that rare germline variants and RNA-specific events cannot be completely excluded by this approach alone. Orthogonal DNA sequencing and comparison with population-level germline and RNA-editing databases will be important for definitive characterization of individual candidate alleles.

By integrating bulk and single-cell RNA sequencing from matched cancer and benign epithelial populations derived from the same patients, we demonstrate that transcriptome-guided variant analysis can identify cancer-associated transcriptional alleles that warrant further genomic validation while resolving the burden of detected transcriptional alleles across defined cellular states. This cancer–benign matched strategy—commonly referred to as patient-matched tumor–normal analysis—provides an important approach for reducing the misclassification of inherited variants as tumor-associated signals [33]. Variants detected in cancer cells but not in matched benign epithelial cells were therefore classified operationally as cancer-associated transcriptional alleles, while recognizing that they may include somatic alterations as well as other RNA-specific or technical events that require additional validation. These candidate cancer-associated alleles identified by RNA-seq may serve as molecular markers for assessing the cancerous status of primary cultured 2D prostate cancer cells and 3D cancer spheroids. Because clinical tumor specimens often contain a heterogeneous mixture of malignant and non-malignant cells [34], confirming that isolated epithelial cells are truly tumor-derived remains a significant challenge. An important strength of our study is the use of a patient-matched cancer–benign design, in which each patient serves as their own control [35]. This approach minimizes the influence of inter-patient genetic variability, reduces the likelihood of misclassifying inherited germline variants as tumor-associated transcriptional alleles, and provides an internal biological control for identifying transcriptional alleles preferentially detected in tumor cells. To further clarify our analytical approach, we have expanded the Methods Section to describe the asymmetric filtering criteria used for novel allele identification, with more stringent thresholds applied to tumor samples and less stringent thresholds applied to matched benign samples. This strategy was designed to minimize false-positive discoveries arising from differences in transcript abundance and sequencing coverage while maximizing confidence that the detected transcriptional alleles are preferentially associated with tumor cells [36]. Furthermore, the cancer–benign matched comparison provides complementary molecular evidence that tumor-derived epithelial cells are molecularly distinct from their benign counterparts.

In the current study, across all patients examined, most cancer-associated variants occurred within noncoding regions, particularly the 3′ and 5′ untranslated regions (UTRs) of expressed transcripts. While this distribution partially reflects the expression bias inherent to RNA-seq–based variant detection, it also suggests that regulatory alterations affecting post-transcriptional control may be more prevalent than coding variants in these tumor populations. Growing evidence indicates that sequence variation within UTRs can influence mRNA stability, localization, and translational efficiency, thereby modulating gene expression independently of coding sequence changes [37]. The enrichment of UTR variants observed here, therefore, highlights a potentially underappreciated regulatory layer in prostate cancer biology that warrants further functional investigation.

Among the 223 genes harboring cancer-associated variants, several—including CITED2, YOD1, MCM4, HNRNPA2B1, KIF20B, DPYSL2, and NR4A1—were significantly dysregulated within cancer stem cell (CSC) populations. A subset of these genes also contained predicted damaging alterations: 19 genes carried non-synonymous variants, and 11 met criteria for deleterious impact based on SIFT prediction, frameshift events, stop-gain or stop-loss mutations, or insertion–deletion events [38]. Particularly notable were alterations in ATF6 and KDM3A, genes implicated in endoplasmic reticulum stress signaling and androgen receptor–associated chromatin regulation, respectively [25,26]. Although the present study was not designed to establish causal driver status, the convergence of mutational and transcriptional perturbations within these pathways suggests that they may represent functionally relevant nodes within prostate cancer regulatory networks.

Cancer stem cells (CSCs) represent a functionally distinct population of tumor cells with self-renewal and tumor-propagating properties and have been implicated in prostate cancer progression, treatment resistance, and disease recurrence. Previous studies, including our own single-cell analyses, have identified distinct transcriptional programs associated with CSC populations in prostate cancer [11,17]. However, whether CSC-enriched populations exhibit distinct patterns of expressed sequence variation remains poorly understood. Characterizing transcriptional alleles in CSC and non-CSC populations may provide an additional molecular perspective on the biology of these cells. Single-cell analysis further revealed that the burden of detected transcriptional alleles is not uniformly distributed across tumor cell populations. CSC-specific compartments consistently exhibited fewer coding and noncoding variants than non-stem cancer cells in both 2D cultures and 3D spheroids. The differences in the detection of RNA-derived transcriptional alleles among cellular populations may reflect variation in transcriptional activity, transcript abundance, gene coverage, sequencing depth, allele expression, or single-cell dropout, in addition to potential underlying biological differences. Several non-exclusive mechanisms may contribute to this observation, including more efficient DNA damage response pathways, fewer cumulative replication cycles, or selective evolutionary pressures that preserve genome stability in long-lived tumor-initiating cells. Comparable protective patterns have been described in normal tissue stem cells and in certain malignancies, supporting the biological plausibility of this interpretation [39,40].

Differences between conventional 2D cultures and CSC-enriched spheroids further underscore the influence of cellular composition and culture context on the measurable burden of detected transcriptional alleles. The higher variant counts detected in bulk 2D cancer cell populations likely reflect expansion of more differentiated progeny, increased transcriptional complexity, or technical differences in transcriptomic coverage. These findings emphasize the importance of interpreting transcriptional allele landscapes derived from bulk sequencing approaches within the context of underlying cellular heterogeneity [41]. At the same time, differences in the number of RNA-derived alleles detected between cellular populations may reflect biological and technical factors, including differences in transcript abundance, transcriptional activity, sequencing coverage, and single-cell dropout. Therefore, differences in detected transcriptional alleles should not be interpreted directly as differences in genomic mutation burden or genome stability without independent DNA-based evidence.

Taken together, our results suggest the existence of a hierarchical gradient of transcriptional alleles within prostate tumors, in which CSCs represent a relatively genomically safeguarded reservoir capable of sustaining long-term tumor maintenance. The reduced burden of detected transcriptional alleles observed in CSCs may reflect intrinsic genome-maintenance programs inherited from long-lived progenitor populations, potentially contributing to their persistence and tumor-propagating capacity. If confirmed by DNA-based sequencing and functional studies, such mechanisms may represent an important vulnerability: disruption of CSC-specific genome-stability pathways could compromise the cellular reservoir that fuels tumor recurrence and therapeutic resistance [30].

More broadly, this study illustrates the utility of transcriptome-derived variant analysis combined with single-cell resolution for interrogating somatic variation within defined tumor cell states. Identification of cancer-associated alleles, together with the transcriptional alleles’ gradient observed across the prostate cancer hierarchy, suggests that CSC-focused vulnerabilities may complement existing androgen receptor–directed therapeutic strategies. Future studies integrating matched DNA sequencing, larger patient cohorts, and mechanistic interrogation of candidate genes such as ATF6 and KDM3A will be required to determine the extent to which the alterations identified here contribute to prostate cancer initiation, progression, and therapeutic resistance.

Finally, we acknowledge that RNA-derived transcriptional alleles may arise from multiple biological and technical sources, including expressed somatic mutations, germline variants, RNA editing, allele-specific expression, alternative splicing, and sequencing or mapping artifacts [36]. We also recognize that the present analyses were based on RNA sequencing data and were not normalized for transcript length, sequencing depth, or transcript coverage. Therefore, differences in the number and distribution of detected transcriptional alleles between datasets should be interpreted as descriptive observations that may reflect both biological and technical factors rather than absolute measures of genomic mutational burden. To further address these limitations, we performed additional analyses to evaluate the potential contribution of germline variants and RNA editing by annotating the identified RNA-derived transcriptional alleles against the public databases dbSNP, gnomAD, and REDIPortal [18,19,20,21]. Based on these annotations, we estimate that only a minority of the detected transcriptional alleles overlap with known germline variants or annotated RNA-editing sites. Interestingly, the single-cell RNA-seq dataset showed a higher proportion of overlap with RNA-editing sites than the bulk RNA-seq dataset, suggesting that differences in RNA editing may, at least in part, contribute to the transcriptional diversity observed among individual cancer cell clusters. Accordingly, the transcriptional alleles identified in this study should not be interpreted uniformly as somatic genomic mutations; rather, they represent RNA-derived allelic signals preferentially detected in cancer cells, some of which may reflect somatic mutations, rare germline variation, RNA editing, allele-specific expression, or other transcriptomic processes. The primary objective of the present study was to identify candidate cancer-associated RNA-derived transcriptional alleles that warrant further investigation, rather than to establish their underlying genomic origin. Orthogonal DNA-based validation will therefore be required to distinguish expressed somatic mutations from germline variants, RNA-editing events, and other RNA-derived signals, and to confirm bona fide somatic mutations. We emphasize that the findings of this study are exploratory and hypothesis-generating, providing a foundation for future genomic validation, multi-omics integration, and functional investigation.

## 5. Conclusions

In conclusion, this study demonstrates the potential of patient-matched bulk and single-cell RNA sequencing to identify cancer-associated RNA-derived transcriptional alleles and to characterize their distribution across distinct cellular states in prostate cancer. We identified 223 genes harboring cancer-associated transcriptional alleles, with a predominant enrichment in noncoding UTRs, highlighting a potentially important regulatory dimension of prostate cancer biology. Several variant-bearing genes, including CITED2, YOD1, MCM4, HNRNPA2B1, KIF20B, DPYSL2, NR4A1, ATF6, and KDM3A, were associated with cancer stem cell states or potentially functional alterations, suggesting candidate pathways that may contribute to tumor maintenance and progression. Notably, CSC populations exhibited a lower burden of detected transcriptional alleles than non-stem cancer cells in both 2D cultures and 3D spheroids, supporting a potential hierarchical pattern in which tumor-propagating cells maintain relatively greater genome stability. However, because RNA-derived alleles may reflect expressed somatic mutations, germline variation, RNA editing, allele-specific expression, alternative splicing, or technical artifacts, these findings should be interpreted as exploratory and hypothesis-generating rather than definitive measures of genomic mutational burden. Overall, our findings provide a framework for investigating transcriptional allele heterogeneity within the prostate cancer cellular hierarchy and suggest one possible biological solution that CSC-associated genome-maintenance mechanisms may represent potential therapeutic vulnerabilities. Matched DNA sequencing, larger patient cohorts, and functional validation of candidate alterations will be essential to establish the genomic origin, biological significance, and therapeutic relevance of these transcriptional alleles.

## Figures and Tables

**Table 1:** Common non-synonymous variants in cancer cells.

Gene Name	Description
ANKRD36C	Ankyrin Repeat Domain 36C
ATF6	Activating Transcription Factor 6
CCDC186	Coiled-Coil Domain Containing 186
CDS1	CDP-Diacylglycerol Synthase 1
FAT1	FAT Atypical Cadherin 1
GIPC1	GIPC PDZ Domain Containing Family, Member 1
GOLGB1	Golgin B1
HDGF	Heparin-Binding Growth Factor
HLA-C	Major Histocompatibility Complex, Class I, C
KDM3A	Lysine Demethylase 3A
KIF20B	Kinesin Family, Member 20B
MAST4	Microtubule-Associated Serine/Threonine Kinase Family, Member 4
MTRF1L	Mitochondrial Translation Release Factor 1 Like
NPIPB5	Nuclear Pore Complex Interacting Protein Family, Member B5
NR4A1	Nuclear Receptor, Subfamily 4, Group A, Member 1
PRCC	Proline-Rich Mitotic Checkpoint Control Factor
RP11-2C24.9, SRCAP	Snf2-Related CREBBP Activator Protein
SMARCA5	SWI/SNF-Related, Matrix-Associated, Actin-Dependent Regulator of Chromatin, Subfamily A, Member 5
ZFPL1	Zinc Finger Protein Like 1

**Table 2:** Common non-synonymous variants with damaged protein functions in cancer cells.

Gene Name	Description
ATF6	Activating Transcription Factor 6
CDS1	CDP-Diacylglycerol Synthase 1
FAT1	FAT Atypical Cadherin 1
GOLGB1	Golgin B1
HDGF	Heparin-Binding Growth Factor
KDM3A	Lysine Demethylase 3A
MAST4	Microtubule-Associated Serine/Threonine Kinase Family, Member 4
MTRF1L	Mitochondrial Translation Release Factor 1 Like
NPIPB5	Nuclear Pore Complex Interacting Protein Family, Member B5
PRCC	Proline-Rich Mitotic Checkpoint Control Factor
RP11-2C24.9, SRCAP	Snf2-Related CREBBP Activator Protein

**Table 3:** Total number of novel alleles per variant location/type in cancer stem cells (CSCs) vs. non-CSCs from both 2D and 3D cultures.

Cell Culture Type	Exonic	Noncoding	Indel or Complex	SNV	Non-Synonymous _SNV	Synonymous _SNV	Non-Frameshift _Insertion	Stop-Gain
2D	79	1813	106	1786	62	15	0	2
3D	10	537	44	503	5	4	1	0
2D_CSC	2	59	1	60	1	1	0	0
3D_CSC	0	15	0	15	0	0	0	0

**Table 4:** Seven genes were significantly dysregulated in cancer stem cells (CSCs) versus non-CSCs.

Gene Name	CSC/Non-CSC Fold Changes
CITED2	16.610
YOD1	8.867
MCM4	−4.056
HNRNPA2B1	−4.168
KIF20B	−5.069
DPYSL2	−6.027
NR4A1	−6.183

**Table 5:** Possible germline and RNA-editing origins of novel alleles detected. SNVs were characterized based on minor allele frequency (MAF) > 1% in gnomAD. Rare SNVs were characterized based on MAF < 1% and > 0% in gnomAD or annotated in dbSNP. The fraction of sites overlapping with REDIPortal is also shown.

	Bulk RNA-Seq		Single-Cell RNA-Seq	
Germline Origin	Total	Overlap with REDIPortal	Total	Overlap with REDIPortal
SNV (MAF > 1%)	8337 (31.8%)	74 (0.2%)	63 (3.2%)	1 (0.05%)
Rare SNV (MAF < 1%)	2715 (10.3%)	47 (0.3%)	238 (12.2%)	50 (2.6%)
Uncharacterized	15,192 (57.9%)	247 (0.9%)	1685 (84.6%)	235 (12.0%)

## Data Availability

The original contributions presented in this study are included in the article/Supplementary Materials. Further inquiries can be directed to the corresponding authors.

## Supplementary Materials

The following supporting information can be downloaded at: https://www.mdpi.com/article/10.3390/biom16091341/s1, Supplemental Table S1. A total of 223 genes harboring RNA-derived unique exonic or UTR variants were detected exclusively in cancer cells across patients.

## Abbreviations

The following abbreviations are used in this manuscript:

RNA-seq	RNA sequencing
SNV	Single-nucleotide variant
CSCs	Cancer stem cells
UTRs	Untranslated regions
PrE-Ca	Prostate cancer epithelial cells
PrE-pz	Benign cells from the prostate peripheral zone
PrEGM	ProstaLife Epithelial Cell Growth Medium
PCA	Principal component analysis
GOI	Genes of interest
UMAP	Uniform Manifold Approximation and Projection
SIFT	Sorting Intolerant From Tolerant
