# Supplementary material for: Identification of Novel Transcriptional Alleles in Primary Prostate Cancer Cells and Cancer Stem Cells by Bulk RNA-Seq and Single-Cell RNA-Seq Analyses
Source: Biomolecules. 2026 Sep 15;16(9):1341. doi: 10.3390/biom16091341 (PMC13604742; doi:10.3390/biom16091341)
Supplement: Supplementary file 1 [file biomolecules-16-01341-s001.zip › biomolecules-4520401-supplementary.pdf]

Supplemental Table S1. A total of 223 genes harboring RNA-derived unique exonic or UTR variants were detected exclusively in cancer cells across patients.

ACER3	ENAH	NSD3	SOX4
AHNAK2	ESCO1	OIP5-AS1	SPSB1
AK4	EXOSC6	P4HB	SPTY2D1
AKAP11	FAM172A	PAPD5	STX16
AKAP9	FAM43A	PATJ	STX2
ALDH9A1	FAM83F	PAWR	SULT1A1
AMMECR1	FAT1	PCDH7	SUN1
ANKRD12	FBXO3	PDCL	SZRD1
ANKRD36C	FTX	PEX19	TBC1D8
ANPEP	FURIN	PHAX	TBC1D8B
AP1S2	GALNS	PPIA	TDRKH
ASCC3	GBF1	PPP2R1B	TENM2
ASXL2	GBP1	PPP2R5C	TJP1
ATE1	GIPC1	PPP2R5E	TLN1
ATF6	GNB5	PRCC	TM9SF3
ATF7	GNPTAB	PRKD3	TMEM107
ATG12	GOLGB1	PRRG4	TMEM131
ATP5SL	GPATCH2L	PSMD11	TMEM192
ATXN1	GTF2IRD1	QKI	TMEM248
B3GAT3	GTF3C4	R3HDM2	TMEM41B
BCL11B	HCFC1	RAB29	TMX1
BIRC6	HDGF	RAET1G	TNFSF10
BMPR2	HLA-C	RBM12	TRAP1
BPTF	HNRNPA2B1	RBM17	TRERF1
BRD4	HNRNPK	RBM8A	TRIB2
C1RL	HOOK3	RBPJ	TRMT13
C21orf33	HOXD11	RCAN3	TRUB2
C8orf82	HPS1	RNF144B	TULP3
CASP14	HSPA5	ROBO1	UACA
CCDC186	INPP4B	RP11-147L13.11	UBA6
CCND2	JARID2	RP11-2C24.9,SRCAP	UBE2D3
CCSER2	KCTD10	RPL37A	UBE4B
CDS1	KDM3A	RRBP1	UEVLD
CEBPZOS	KIAA0930	RSF1	UQCC1
CFLAR	KIF20B	RSPH3	USB1
CISD2	KMT5B	SAMD8	USP14
CITED2	KPNB1	SCARB2	USP15
CLCC1,GPSM2	LARS	SDHC	USP53
CLIC4	LCOR	SEC62	VAV3
CNTRL	LINC00969	SEL1L	WAC
CPEB4	LRRC8C	SETX	WASF1
CREBRF	MARVELD2	SFPQ	WDR19
CSNK1G2	MAST4	SFT2D2	XDH
CSNK1G3	MCM4	SLAIN2	XIAP
DCBLD1	METTL2B	SLC25A13	XPOT
DCTN5	METTL8	SLC25A37	YOD1
DIP2A	MMP2	SLC35A1	ZBED5
DNAJC1	MOB1A	SLC35E1	ZC3H18
DPYSL2	MRPL30	SLC4A7	ZFPL1
DST	MTMR2	SLC52A2	ZKSCAN1
EBLN3P	MTRF1L	SLC9A6	ZMYND19
EEA1	MXD4	SLFN5	ZNF267
EFNB1	MYL6	SMARCA5	ZNF365
EGLN1	MYO6	SMC5	ZNF431
EIF4EBP2	NPIPB5	SOCS6	ZNF83
ELOVL6	NR4A1	SON	
